# Sulphation and Hydrolysis Improvements of Bioactivities, and Immuno-Modulatory Properties of Edible *Amanita hemibapha* Subspecies *javanica* (Corner and Bas) Mucilage Polysaccharide as a Potential in Personalized Functional Foods

**DOI:** 10.3390/jof7100847

**Published:** 2021-10-10

**Authors:** Utoomporn Surayot, Sutee Wangtueai, Sangguan You, Charin Techapun, Yuthana Phimolsiripol, Noppol Leksawasdi, Warawut Krusong, Francisco J. Barba, Phisit Seesuriyachan

**Affiliations:** 1 College of Maritime Studies and Management, Chiang Mai University, Samut Sakhon 74000, Thailand; utoomporn.su@cmu.ac.th (U.S.); sutee.w@cmu.ac.th (S.W.); 2 Department of Marine Food Science and Technology, Gangneung-Wonju National University, Gangwon 210-702, Korea; umyousg@gwnu.ac.kr; 3 East Coast Life Sciences Institute, Gangneung-Wonju National University, Gangwon 210-720, Korea; 4 Faculty of Agro-Industry, Chiang Mai University, Chiang Mai 50100, Thailand; charin.t@cmu.ac.th (C.T.); yuthana.p@cmu.ac.th (Y.P.); noppol.l@cmu.ac.th (N.L.); 5 Cluster of Agro Bio-Circular-Green Industry, Chiang Mai University, Chiang Mai 50100, Thailand; 6 Division of Fermentation Technology, Faculty of Food Industry, King Mongkut’s Institute of Technology Ladkrabang, Bangkok 10520, Thailand; warawut.kr@kmitl.ac.th; 7 Department of Preventive Medicine and Public Health, Food Science, Toxicology and Forensic Medicine, Faculty of Pharmacy, Universitat de València, Avda. Vicent Andrés Estellés s/n, 46100 Burjassot, Spain; francisco.barba@uv.es; 8 Advanced Manufacturing and Management Technology Research Center (AM2Tech), Department of Industrial Engineering, Faculty of Engineering, Chiang Mai University, Chiang Mai 50200, Thailand

**Keywords:** mucilage polysaccharide, mushroom, *Amanita hemibapha* subspecies *javanica* (Corner and Bas), immunomodulatory, sulphation, hydrolysis

## Abstract

In this study, the mucilage polysaccharide (MP) from *Amanita hemibapha* subspecies *javanica* was prepared by hot water extraction and ethanol precipitation and then fractionated using anion-exchange chromatography equipped with a DEAE Sepharose fast flow column. The most immune-enhancing polysaccharide fraction 2 (MPF2) was subjected to a structural modification such as hydrolysis or over-sulphation. The sulphate and molecular weight (Mw) of over-sulphated (OS1-3) and hydrolysed (HS1-3) derivatives of MPF2 differed between 9.85% and 14.2% and 32.8 and 88.1 × 10^3^ g/mol, respectively. Further, the immune-enhancing properties of MPF2 and its derivatives were tested on RAW264.7 and NK cells through various in vitro assays. Interestingly, a low molecular weight of HS1-3 significantly increased the nitric oxide (NO) production (*p* < 0.05) more than MPF2, indicating that Mw is a major factor in RAW264.7 cell stimulation. In addition, RAW264.7 cells produced various cytokines by up-regulating mRNA expression levels and the activation of nuclear factor kappa-B (NF-κB) and mitogen-activated protein kinase (MAPK) pathways. On the other hand, OS1-3-treated natural killer (NK) cells induced cytotoxicity in HepG2 cells through the expression of IFN-γ, Grandzyme-B, perforin, NKp30, and FasL. These results demonstrated that sulphate derivatives play an important role in NK cell activation. Further, this study also explores how polysaccharide binds to RAW264.7 and NK cells. MPF2 and HS3 may activate RAW264.7 cells via binding to TLR4 receptors, and OS2 could be activated through the CR3 signalling pathways.

## 1. Introduction

*Amanita hemibapha* subspecies *javanica* (Corner and Bas), an edible wild mushroom, is naturally found in northern and north-eastern Thailand. It belongs to the genus *Amanita* in the subspecies of *Amanita hemibapha*. Being one of the most popular wild mushrooms, native Thai people traditionally consume it. Generally, edible mushrooms are an excellent source of nutritional protein, carbohydrates, fat, vitamins, fibre, and minerals, and have been termed “food of the gods” [1,2,3,4]. It has a distinct flavour and a special sense of taste, making it a very popular food. It is widely consumed as a food in many countries such as Japan, China, and South Korea due to its nutritional value and medicinal use. It contains extraordinary medicinal benefits including the prevention of heart diseases and different types of cancers, the improvement of blood circulation and lowering of blood cholesterol level, anti-ageing properties, immunomodulatory functions, and improvement of renal function [5,6,7,8,9]. Several linear/branched glucans and heteroglycans isolated from edible mushrooms demonstrated antimicrobial, hypotensive, antifungal, anti-inflammatory, antiviral, antibacterial, hepatoprotective, anti-diabetic, hypolipidemic, anticancer, immunological, antithrombotics, and antioxidant properties [7,10,11,12,13,14,15,16,17,18,19,20,21,22].

Macrophages are commonly employed to assess the immunomodulatory effects of bioactive substances because they serve critical roles in immunosurveillance against malignant cells and pathogens, as well as antigen presentation [23,24,25]. Aside from pathogenicity and infections, macrophages may detect several natural molecules, such as proteins, glycosides, and polysaccharides, as stimuli to induce immunological responses through the secretion of several cytokines, such as interleukin 1 beta (IL-1β), tumour necrosis factor alpha (TNF-α), nitric oxide (NO), and chemokines [26]. After macrophages are stimulated by pathogens and microorganisms, they induce nitric oxide synthase (iNOS) to produce NO in terms of pathogen killing [27,28]. For example, macrophages are treated with fungal polysaccharides from a variety of sources, including *Amauroderma rude* [29], *Russula griseocarnosa* [30], *Schizophyllum commune* [31], *Dictyophora indusiate* [25], and others, to determine their immunomodulatory activity. In the human body, natural killer (NK) cells are important immune cells derived from myeloid lymphoid stem cells. NK cells are large granule lymphocytes and serve as the first line to protect the immune system. They act as an imperative connection between innate and adaptive immunity and play an important role in anti-inflammatory responses, tumour surveillance and autoimmune disorder regulation [32]. They contain antitumour, antiviral, and anti-inflammatory properties and the ability to detect and destroy targeting cells. Simultaneously, NK cells can secrete a variety of cytokines and chemokines to regulate the function of other immune cells [33,34]. Additionally, they influence the magnitude and direction of the adaptive immune response against tumours. Thus, the key roles of NK cells are cytotoxicity and the ability to produce different cytokines [35]. 

Early research demonstrated that the physiological function and biological activities of a polysaccharide depend upon its molecular weight (Mw), structure, and functional groups [36]. These included the sugar unit, glycosidic bond to the main chain, and type of polymerization degree of branching [37]. Additionally, the increase in bioactivities is proportionate to the degree of sulphation [38]. An over-sulphated glucan possesses higher inhibitory activity against H-22 tumour cells [39]. Moreover, sulphated polysaccharides with different degrees of substitution are involved in antitumor proliferation [40]. The over-sulphation of polysaccharides was found to be more effective antioxidant agents as they improved the superoxide free radical scavenging activity more than native polysaccharides [41]. It showed that the sulphated polysaccharides could promote the production of cytokines and the release of NO, therefore significantly increasing the splenic lymphocyte activation [42]. A recent investigation uncovered that the Mw of polysaccharides directly relates to its immune activity. Researchers found that glucuronoxylo-rhamnans with low molecular weight from 17.3 × 10^3^ to 33.3 × 10^3^ g/mol, indicated the most potent immunostimulant activities [43]. Polysaccharides with an Mw of 38.5 × 10^3^ g/mol are potent, natural, innate immunomodulators with a broad spectrum of bioactivities and immunosuppressive properties, exposing the relationship between its Mw and immunomodulatory activities [44]. Consequently, a polysaccharide’s biological activities and pharmacological activities are linked intimately to its molecular structures [45]. However, the bioactivities need to be improved because some natural or crude polysaccharides exhibited low bioactivity. 

Our prior study examined how the mucilage polysaccharide fraction 2 (MPF2) isolated from *Amanita hemibapha* subspecies *javanica* significantly activated RAW264.7 cells to release NO and different cytokines, as well as being strong immunostimulant agents [46]. In this present study, we evaluated the effector activity of MPF2 polysaccharides, and further investigated molecular structure and the immunomodulatory relationships. The immune-enhancing activity using RAW264.7 cells and NK cells was performed after the sulphate contents and molecular weight modification.

## 2. Materials and Methods

### 2.1. Reagents and Chemicals

The media and other materials required for culturing cells were purchased from Lonza Inc. (Walkersville, MD, USA). Griess reagent and lipopolysaccharide (LPS: *E. coli*, serotype O111:B4; L2630) were purchased from Sigma–Aldrich (St. Louis, MO, USA). The WST-1 assay kit was obtained from Daeillab Service Co., Korea. Anti-Toll-like receptor 2 antibody (anti-TLR2), anti-complement receptor 3 antibody (anti-CR3), and anti-Toll-like receptor 4 antibody (anti-TLR4), were obtained from Abcam (Cambridge, MA, USA). Phospho-NF-κB antibody, phospho-p38 (MAPK) antibody, phospho-ERK (MAPK) antibody, and phospho-JNK antibody were purchased from Cell Signalling Technology (Danvers, MA, USA). All chemicals and reagents used in this work were of analytical grade.

### 2.2. Cell Lines

Macrophage RAW264.7 cells, natural killer cells (NK-92 cells), and hepatocellular carcinoma cell lines (HepG2) were obtained from the American Type of Culture Collection (ATCC, Rockville, MD, USA).

### 2.3. Extraction and Fractionation of Polysaccharide

**Figure 1:**
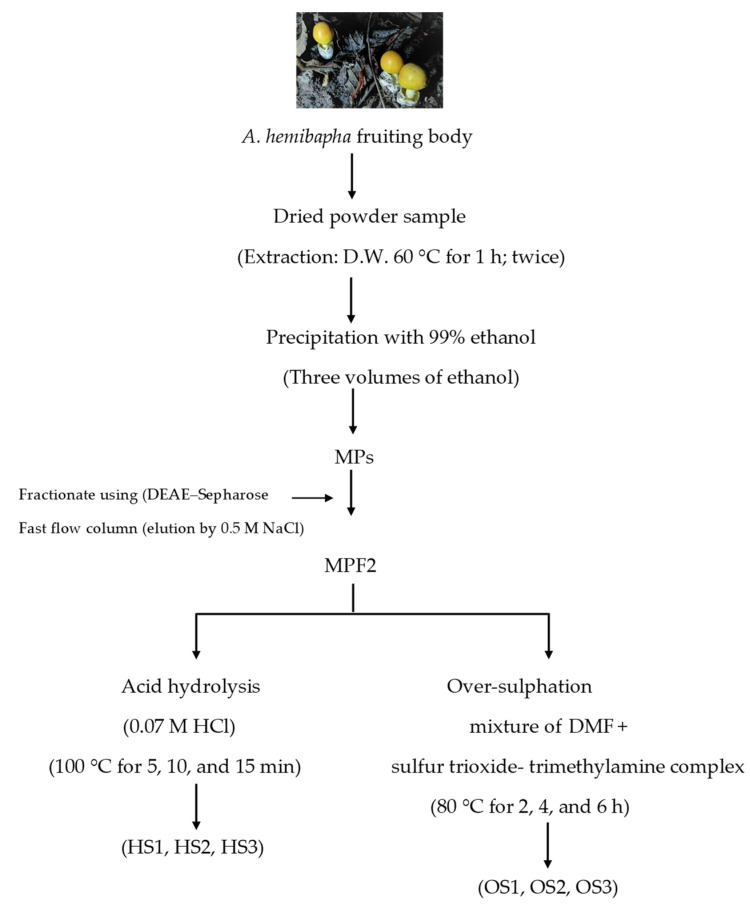
Extraction of polysaccharide (MP) and MPF2 modification process.

The extraction and fractionation of *A. hemibapha* polysaccharide were carried out using the methods described in our previous study [46]. The dried powder sample of *A. hemibapha*’s fruiting body was extracted twice with distilled water at 60 °C for 1 h. The crude polysaccharide was recovered by precipitation with 99% (*v*/*v*) ethanol and then subjected to filtration. The crude sample was fractionated using an ion-exchange chromatography equipped with a DEAE Sepharose fast flow column (17-0709-01; GE Healthcare Bio-Science AB, Uppsala, Sweden). The chromatography yielded two fractions (MPF1 and MPF2), while the most immunostimulant fraction (MPF2) was selected to study the structure–bioactivity relationships (Figure 1). 

### 2.4. Preparation of MPF2 Derivatives

The derivatives from MPF2 that comprised three different levels of average molecular weight and sulphates were prepared under the different experimental conditions. The over-sulphated derivatives were prepared by treating MPF2 with a mixture of dimethylformamide and a sulphur trioxide-trimethylamine complex. The reaction was carried out at 80 °C for 2, 4, and 6 h and then cooled down. Afterwards, the reaction was mixed with a saturated solution of sodium acetate in ethanol and poured into cold ethanol. The mixture was then centrifuged, and the collected sulphated polysaccharides were re-dissolved in distilled water, dialyzed, and lyophilized. The degree of substitution (DS) was calculated from the sulphur content based on Schöniger’s formula [47].

(1) $$DS=\frac{1.62\times S\%}{32-\left(1.02\times S\%\right)}\;$$

The functional groups were identified by FT-IR spectral analysis using a Tensor 27 spectrophotometer (Bruker, Karlsruhe, Germany).

Next, the hydrolysis derivatives were prepared by mixing the sample with 0.07 M HCl, and the reaction was set to react for 5, 10, and 15 min to obtain three different molecular weights. Once the reaction mixture was cooled, the sample was neutralized using 0.07 M NaOH, dialyzed using a cellulose membrane (molecular weight cut off 3,500 Da), and then lyophilized. 

### 2.5. Analytical Methods

The sulphate content of polysaccharides was estimated by the BaCl_2_ gelatine method [48], respectively. The average *Mw* of the polysaccharides was calculated using a high-performance size exclusion chromatography column coupled to UV, multi-angle laser light scattering, and refractive index detection (HPSEC-UV-MALLS-RI) according to the method of Cao et al. [49].

### 2.6. Macrophage Proliferation and Nitric Oxide Production Assay

In this study, cells proliferation was accessed by WST-1 calorimetric assay. RAW264.7 cells were plated in the 96-well microplates at a cell density of 1 × 10^6^ cells/well in a volume of 100 µL. After, the cells were treated with 100 µL of sample solution for 24 h. Over the incubation period, 100 µL of WST-1 solution was introduced into the well and extended the incubation for an additional hour. The absorbance was measured at 450 nm using a microplate reader (EL-800; BioTek Instruments, Winooski, VT, USA).

(2) $$Cell\;proliferation\;\left(\%\right)=\frac{Absorbance\;of\;the\;experimental\;group}{Absorbance\;of\;the\;control\;group}\;$$

Similarly, the level of NO production was measured using the Griess reaction [50]. The release of NO content in the RAW264.7 cell culture media was calculated using the standard curve obtained with NaNO_2_ (1–200 µM in culture medium). 

### 2.7. NK Cell Activity Assay

Likewise, the NK cell proliferation was examined by WST-1 colorimetric assay. The cells were incubated with MPF2 and its derivatives (200 µg/mL) for 24 h at 37 °C. After, the cells were treated with 110 µL of 10% WST-1 solution and then read the absorbance at 450 nm. In the cytotoxicity assay, HepG2 cells were used as the target cells. The stimulated NK cells (7.5 × 10^5^ cells/well) were allowed to co-culture with HepG2 cells at an effector/target ratio of 30:1. This experiment was conducted in triplicate. The plate was incubated at 37 °C in a 5% CO_2_ atmosphere for 4 h. The cytotoxic activity was assessed by WST-1 assay. The percentage of cytotoxicity was calculated using the following formula:

(3) $$\mathrm{Cytotoxicity}\;\left(\%\right)=\left(1-\frac{\mathrm{Abst}}{\mathrm{Absc}\;}\right)\times 100$$

Where Abst represents the average OD450 of NK cells test, Absc represents the average OD450 of NK cells control. 

### 2.8. Gene Expression by RT-PCR 

RAW264.7 and NK cells were seeded in a 24-well plate at a density of 1 × 10^6^ cells/well and incubated with samples. After 24 h, the total RNA from the cells was extracted using TRIzol reagent (Invitrogen, Carlsbad, CA, USA). The concentration of RNA was measured with a spectrophotometer before constructing cDNA with an oligo-(dT) 20 primer and Superscript III RT (Invitrogen). The resulting cDNA was amplified by PCR using GoTaq Flexi DNA Polymerase (Promega, Madison, WI, USA). The PCR product gels were viewed under UV transillumination. The nucleotide sequences of the primers are shown in Table 1.

### 2.9. Western Blot Analysis

Western blotting was performed according to standard procedure. RAW264.7 cells were seeded at 1 × 10^6^ cells/well in a 6-well plate, and then treated with LPS or samples for 6 h. The RAW264.7 cells were lysed in RIPA buffer (Tech, and Innovation, Chuncheon, Gangwon, South Korea) containing Inhibitor Cocktail (HaltTM protease, and phosphatase). Cell lysates were separated through 10% SDS-PAGE and transferred onto PVDF membranes. The membranes were subsequently blocked in 5% (*w*/*v*) non-fat skimmed milk (prepared in Tris-buffered saline containing Tween-20, TBST) at room temperature for 1 h and incubated with primary antibodies including tubulin (1:1000), anti-phospho-NF-κB (1:1000), anti-phospho-ERK (1:1000), anti-phospho-p38, and anti-phospho-JNK (1:1000) at 4 °C overnight. The membranes were washed with TBST and incubated with HRP-conjugated anti-rabbit antibody for 1 h at room temperature. Protein was detected using the Pierce^®^ECL Plus Western Blotting Substrate (Thermo Scientific, Waltham, MA, USA) in accordance with the manufacturer’s instructions (Table S1). The bands were visualized using Image Lab software under a ChemiDocTM imaging system.

### 2.10. Cell Binding Receptor

The mechanism for the NK and RAW264.7 cells activation process was investigated by blocking cell surface receptors, such as TLR2, TLR4, and CR3. The cells were pre-incubated with or without the presence of antibodies such as anti-TLR2, anti-TLR4, anti-CR3 (25 µg/mL) for 2 h separately before the polysaccharide treatment. The experiment was performed the same as the level of NO from the RAW264.7 cells, and cytotoxicity assay from NK cells described prior.

### 2.11. Statistical Analysis

The statistical analysis was performed using SAS software (SAS Institute, Cary, NC, USA). All the experiments were performed in triplicate (n = 3) and data were recorded as the mean value with standard deviation (SD). Statistical differences were tested using one-way analysis of variance (ANOVA). Further, multiple comparisons used the LSD test to evaluate the significant difference between groups. Statistical significance was defined as *p* < 0.05.

## 3. Results and Discussion

### 3.1. Preparation of MPF2 Derivatives

The crude polysaccharide was extracted from *A. hemibapha* and subsequently fractionated by ion-exchange chromatography, which yielded two fractions, MPF1 and MPF2, containing different ionic strengths. Between the two fractions, MPF2 showed higher NO production and cytokine-releasing capacity in RAW264.7 cells, implying that it has potent immunostimulant properties. MPF2 mainly consisted of carbohydrates (83.5%), along with considerable amounts of protein (7.2%) and sulphate (9.3%). The monosaccharide compositions of MPF2 were glucose (98.4%), galactose (0.2%), mannose (0.3%), arabinose (0.3%), and rhamnose (0.8%) [46]. Glycosidic linkage analysis showed that MPF2 was mainly composed of a backbone of α-D-(1→6)-glucopyranoside.

**Figure 2:**
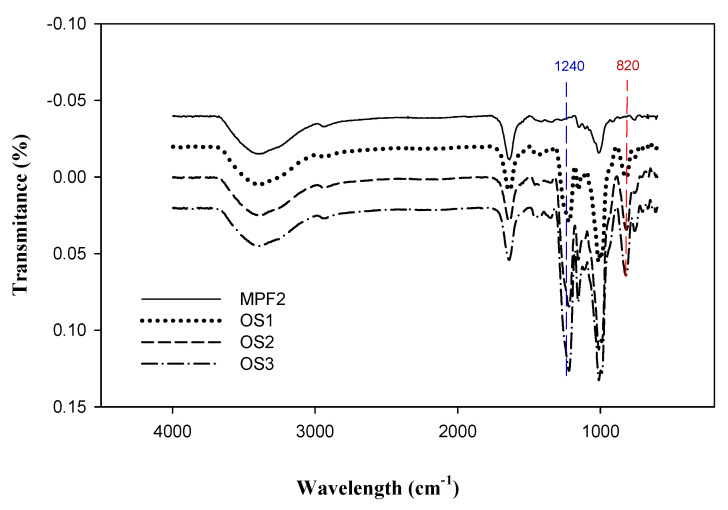
FT-IR spectra of MPF2 and its over-sulphated polysaccharide at 80 °C for 2 h (OS1), 4 h (OS2), and 6 h (OS3).

**Figure 3:**
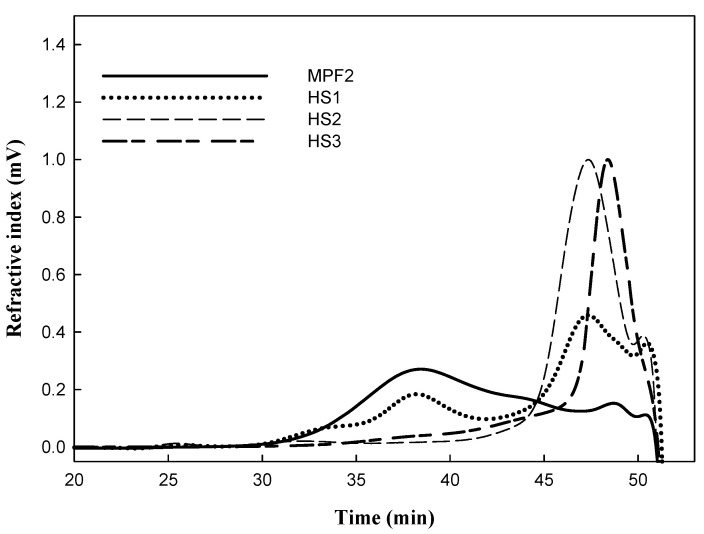
The HPSEC chromatograms of MPF2 and hydrolysed polysaccharides at 100 °C for 5 min (HS1), 10 min (HS2) and 15 min (HS3).

Table 2 and Table 3 show the proximate composition of MPF2 as being a combination of over-sulphated polysaccharide (OS1-3) and hydrolysed polysaccharide (HS1-3). As shown in Table 2, the sulphate content was markedly elevated from 9.30% to 9.85% after 2 h of over-sulphation, and further significantly increased to 11.3% and 14.2% after 4 and 6 h, respectively. The calculated DS values of OS1-3 also significantly increased with reaction times from 0.72 to 0.89 and 1.31, respectively. These results are similar to the data reported by Han et al. [51] and Gunasekaran et al. [52]. The increase in sulphation levels was also shown in the FT-IR spectrum (Figure 2). Compared to MPF2, two characteristic absorption bands at 1240 cm^−1^ and 820 cm^−1^ in the OS1, 2, 3 spectra successively increased in parallel with sulphation time. These results indicated that the sulphate esters were successfully substituted to MPF2 by over-sulphation. However, the over-sulphated reaction led to a slight decrease in the Mw of MPF2 from 104 × 10^3^ g/mol to 98.1, 95.5 and 92.1 × 10^3^ g/mol after 2, 4 and 6 h of reaction time. The molecular degradation seemed inevitable due to the heat treatment during the reaction [53]. Therefore, to investigate the effect of Mw on the bioactivity, the lower polysaccharides (HS1, 2, and 3) were obtained by acid hydrolysis of MPF2 using 0.07 M HCl for 5–15 min. The significant changes in the Mw of MPF2 are shown in HPSEC chromatograms (Figure 3), in which MPF2 was eluted from the SEC columns between 32 and 51 min. After 5 min of hydrolysis, the elution time of the major peak slightly increased to 35–51 min. However, when treated with acid for 10 and 15 min, the elution peaks significantly shifted to 43–51 min and 47–51 min, showing marked degradation of polysaccharides. Table 3 also showed that the chemical gradation by acid hydrolysis treatment significantly decreased the Mw of MPF2 from 104 × 10^3^ g/mol to 88.1 × 10^3^, 50.7 × 10^3^, and 32.8 × 10^3^ g/mol for HS1, 2, 3, respectively. The degradation of polysaccharides was consistent with the result of Table 3, also shown by Xu et al. [54]. These results revealed that it was possible to obtain MPF2 derivatives with different amounts of sulphates as well as different Mw. Therefore, the MPF2 derivatives enabled us to investigate the immunostimulatory effects of various sulphates and Mw of MPF2 on RAW264.7 and NK cells.

### 3.2. Effects of MPF2 Derivatives on RAW264.7 Cell Activation

**Figure 4:**
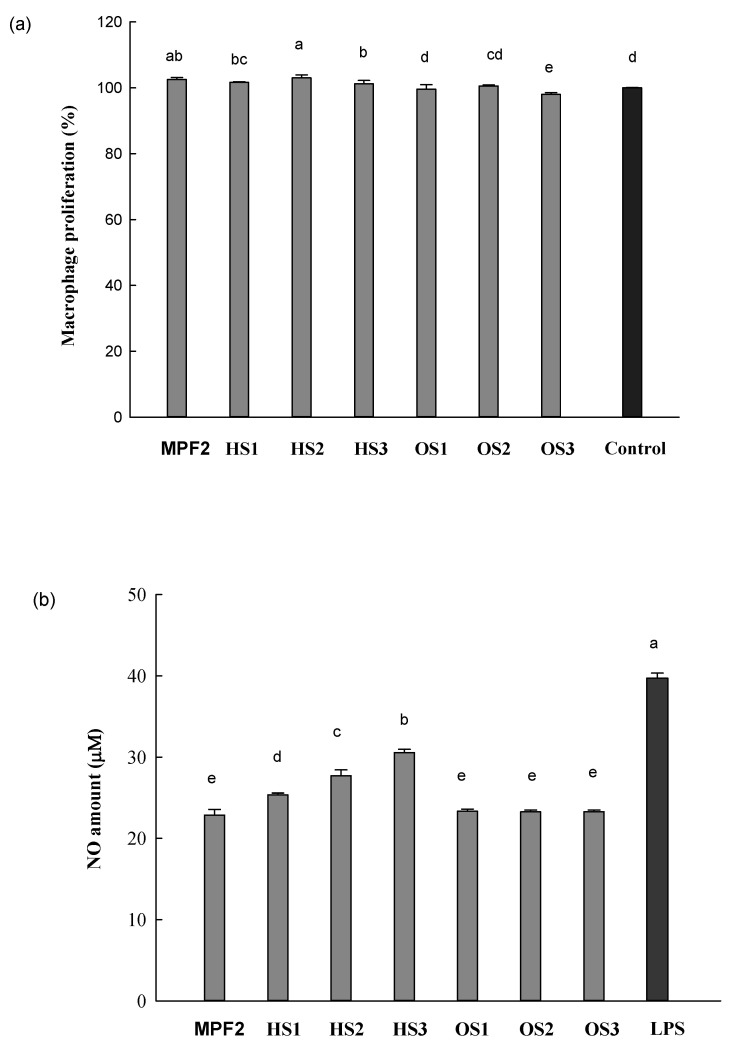

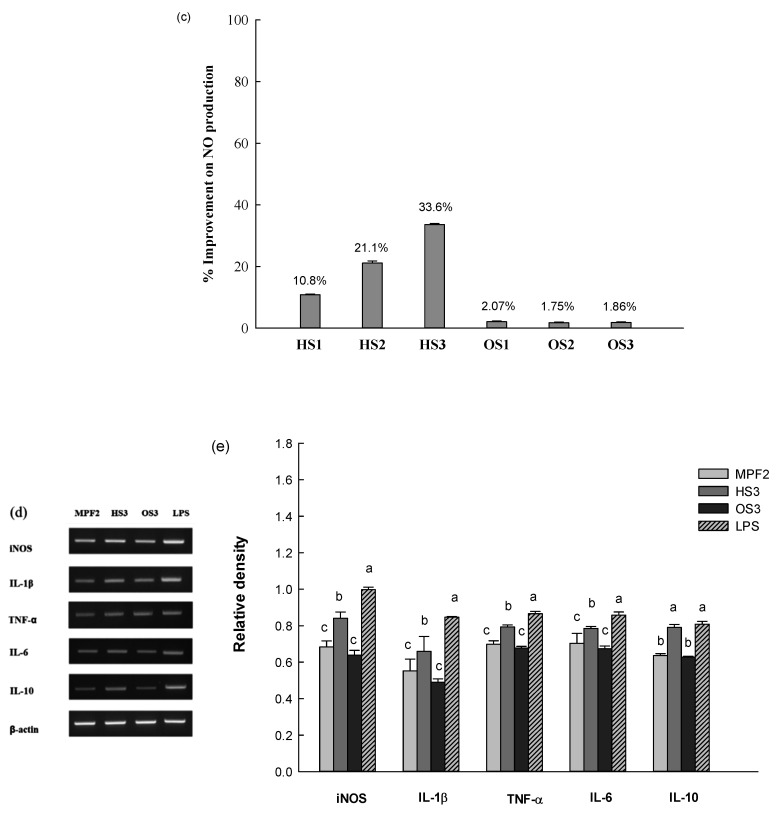
Effect of MPF2 and derivatives on RAW264.7 cells activities: (**a**) cell proliferation and (**b**) nitric oxide (NO) production in RAW264.7 cells, (**c**) percentage improvement on NO production, (**d**) mRNA expression in RAW264.7 cells, (**e**) and relative band intensity. Different letters indicate significant differences (*p* < 0.05) between MPF2 and its derivatives at each cytokine.

RAW264.7 cells, a murine macrophage cell line, release immune-related chemokines and cytokines such as NO, prostaglandin E2 (PGE2), and interleukins (IL) when they are activated by LPS [55]. This system has been used to investigate the immunostimulatory activities of compounds by determining the level of released chemokines and cytokines. In this study, the immunostimulant of MPF2 derivatives (HS1, 2, 3 and OS1, 2, 3) was investigated using RAW264.7 cells by determining the NO production. Figure 4a shows the effect of MPF2 and its derivatives (200 µg/mL) on the proliferation of RAW264.7 cells. The cell proliferation did not occur after treatment with MPF2 and its derivatives (HS1, 2, 3 and OS1, 2, 3). This indicates that samples were not toxic to the cells at the tested concentration. Further, the NO-releasing capacities of MPF2 and its derivatives were tested on RAW264.7 cells at the concentration of 200 µg/mL. LPS (1 µg/mL) was used as a positive control. As shown in Figure 4b, the NO content released from RAW264.7 cells by MPF2 was found to be 22 μM; however, the considerably higher levels of NO release by HS1, 2, 3 were 24, 30, and 33 μM. This indicates that the NO production of H3 was comparable to the amount of NO produced by LPS (positive control). Conversely, no increase in NO production was observed by OS1, 2, 3 compared to MPF2. The percentage of enhanced activity shows the increase in NO production related to MPF2. As for the percentage of improvement, the low molecular weight of MPF2 such as HS1, 2, and 3 increased biological activity by 10.8%, 21.1%, and 33.6%, respectively, but shows a very low percentage of improvement on OS1, 2, 3 (Figure 4c). 

However, this trend was not observed in this study which suggests that the sulphate groups in MPF2 were not an important factor for stimulating RAW264.7 cells. Instead, the Mw of MPF2 was found to be related to the NO-releasing capacity of RAW264.7 cells. The HS activated RAW264.7 cells, resulting in the NO induction, which was significantly higher than that of MPF2 (*p* < 0.05). Among the HS1, 2, and 3, the -HS3 group exhibited the highest NO production. This suggests that the optimum range of Mw may exist for the stimulation of RAW264.7 cells. In a study of exopolysaccharide Mw values, ≤70 × 10^3^ g/mol exhibited the most potent macrophage stimulation, in which the plausible optimum Mw was also suggested for the macrophage activation [56]. In addition, low molecular weights exhibiting rigid conformations and high molecular weights exhibiting compact conformations might be the reason why the low molecular weight affected this research [57]. It was reported by Liu et al. [43] that the polysaccharide extracted from *Enteromorpha prolifera* with the Mw of 33.3 × 10^3^ g/mol exhibited significant improvement of the immune system and cyclophosphamide-induced immunosuppression in mouse models. In addition, the results also reported that the crude polysaccharide with Mw less than 200 × 10^3^ g/mol are good immunogens and demonstrate immunostimulation by producing cytokines from macrophages [58]. The low Mw polysaccharides are considerably facilitated with binding affinity of the vascular endothelial growth factor 165 (VEGF165) to its receptor, possibly due to the formation of various bridge types. This suggested that lower Mw polysaccharides might readily possess conformations to enhance their binding capacity [52,59]. It was therefore suggested that HP with a lower Mw have a better binding ability to the cell receptors than high Mw MPF2.

To determine whether the increased levels of NO production by MPF2 and its derivatives were interconnected to the mRNA expression of inducible nitric oxide synthetase (iNOS), RAW264.7 cells were treated with MPF2 and its derivatives (HS3 and OS3) at a concentration of 200 µg/mL. The levels of iNOS mRNA expression were then examined by an agarose gel analysis of the RT-PCR products. As shown in Figure 4d,e, stronger bands were observed in the HS3 treatment than MPF2, and OS3. These findings indicated that HS3 does stimulate iNOS expression. From these results, it can be concluded that the significant increase in NO production is due to the up-regulated iNOS mRNA expression in RAW264.7 cells after stimulation by partially hydrolysed MPF2. The mRNA expression of other cytokines was also examined by agarose gel electrophoresis of the RT-PCR products. A similar trend was observed for the mRNA expression of IL-1β, TNF-α, IL-6, and IL-10 (Figure 4d,e). Overall, HS3 significantly stimulates RAW264.7 cells through the production of cytokines rather than MPF2, proving that hydrolysis of MPF2 is a better method for enhancing bioactivity.

**Figure 5:**
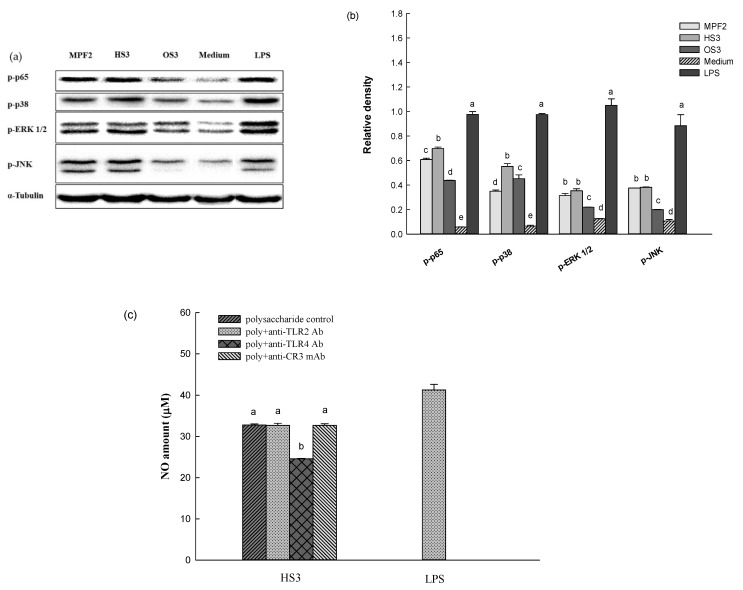
NF-κB and MAPK phosphorylation in RAW264.7 cells: (**a**) the phosphorylation of p65, ERK, P38, and JNK by the treatment of MPF2, HS3, and OS3, (**b**) relative band intensity, (**c**) HS3 on antibody neutralization assay. Different letters indicate significant differences (*p* < 0.05) between polysaccharide control (HS3), and HS3 + anti-TLR2 Ab, HS3 + anti-TLR4 Ab, and HS3 + anti-CR3 Ab.

An additional experiment was carried out to investigate how MPF2 and its derivatives (HS3 and OS3) induced inflammatory mediators. As revealed in Figure 5a,b, the p65 expression was strongly observed upon the treatment of HS3. This indicates that the derivative, HS3, induced the phosphorylation of the p65 subunit from the cytosol to the nucleus. Therefore, it was likely that the phosphorylation of p65 might lead to the activation of nuclear factor kappa-B (NF-κB), which results in the stimulation of the RAW264.7cells. Macrophages are known to be activated not only by the transcriptional activation of NF-κB, but also by the phosphorylation of mitogen-activated protein kinases (MAPK) family members such as ERK, JNK, and p38. Figure 5a,b showed that the treatment of the HS3 derivatives induced the phosphorylation of ERK, JNK, and p38. Overall, these results demonstrate that HS3 stimulates RAW264.7 cells by activating the NF-κB and MAPK pathways.

**Figure 6:**
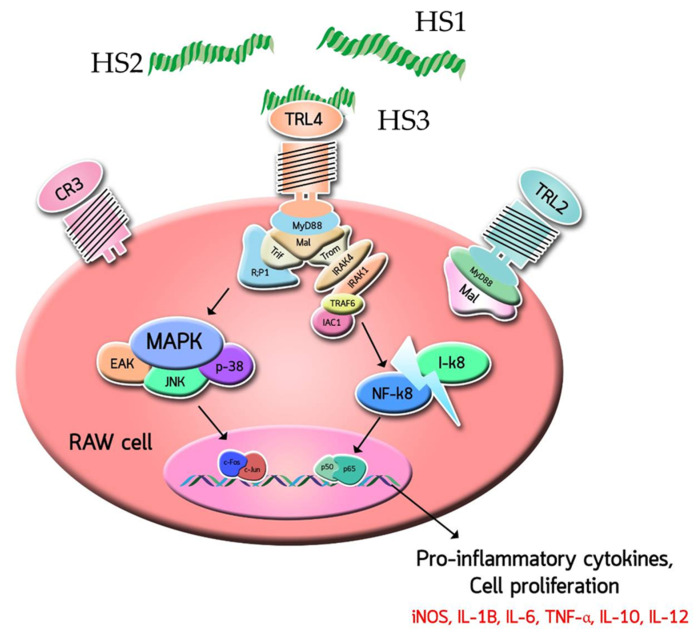
A schematic illustration of the interaction of low molecular weight binding to TLR4.

The involvement of antibodies (anti-TLR2, anti-TLR4, and anti-CR3) in the activation of RAW264.7 cells by HS3 treatment was also studied. The RAW264.7 cells were pre-incubated separately with the respective antibodies, followed by treatments with either MPF2 or its derivatives (HS3 and OS3). This assay was carried out by quantifying the NO released from the RAW264.7 cells. The control sample revealed 41.3% of NO release; however, after pre-incubation with the anti-TLR4, NO released from RAW264.7 cells treated with HS3 was found to be reduced (Figure 5c). Conversely, this trend was not observed with other antibody treatments (anti-TLR2 and anti-CR3). Therefore, these results demonstrate that HS3 derivatives may promote RAW264.7 cells activity via a TLR4 mediated signalling pathway (Figure 6).

### 3.3. Effects of MPF2 Derivatives on NK Cell Activities

NK cells are important cells in the innate immune system by directly eliminating cancer cells and pathogen-infected cells when they are activated by target cell recognition and through signal integration from both activating and inhibitory receptors [32]. Polysaccharides from plants and fungi specifically promote the activation and cytotoxicity of NK cells by enhancing interferon-alpha/beta (IFN-α/γ), granzyme-B, and perforin secretion, and increase the expression of the activating receptor, NKp30 [60]. In this study, the effect of MPF2 and its derivatives on NK cell activation and cytotoxicity was investigated by cytotoxicity assay and the gene expressions of IFN-γ, granzyme-B and perforin, NKp30, and FasL. 

**Figure 7:**
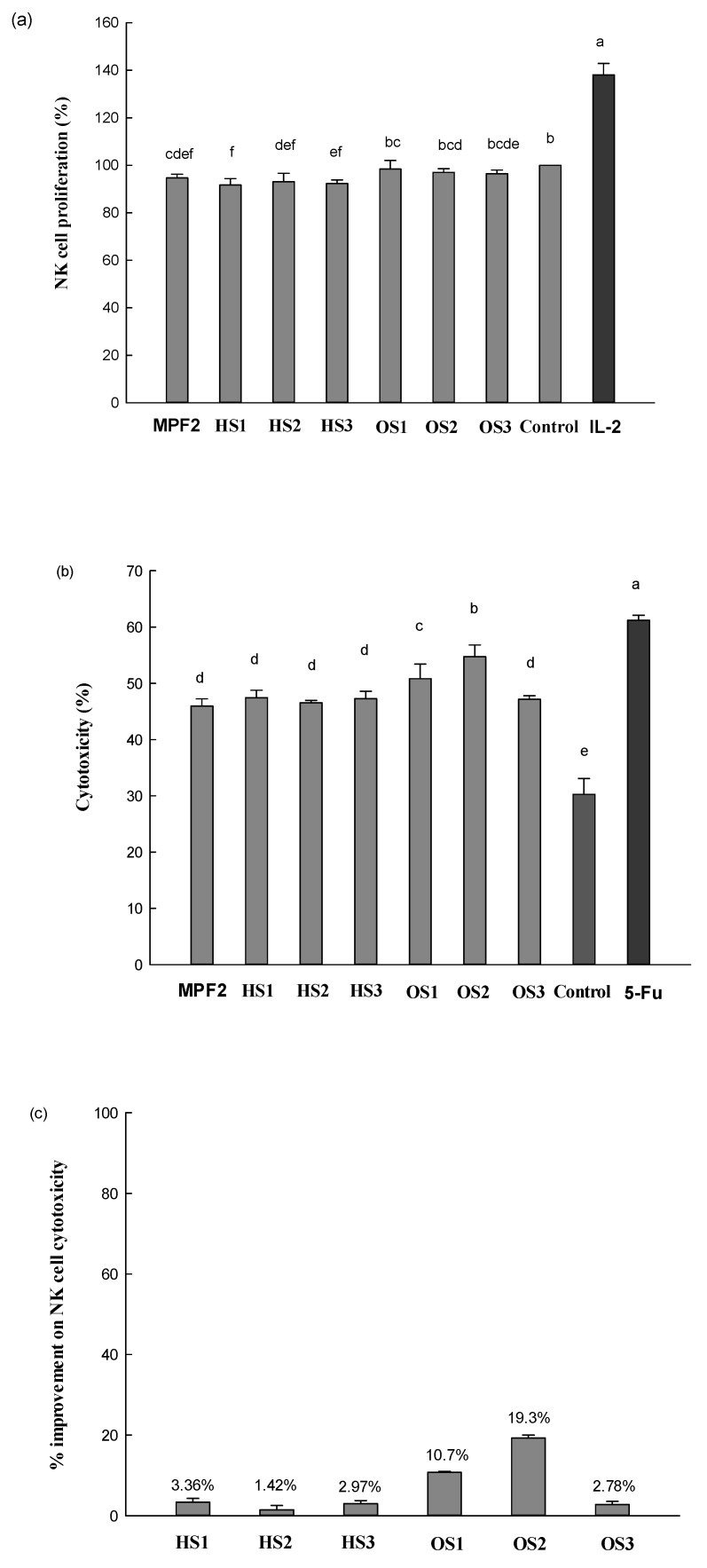

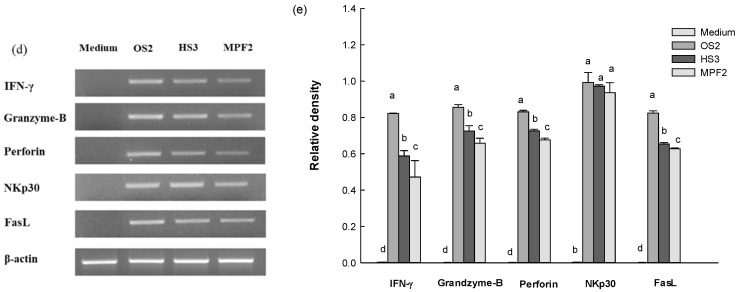
Effect of MPF2 and derivatives on NK cell activities: (**a**) cell proliferation and (**b**) cytotoxicity against HepG2 cells, (**c**) percentage improvement on NK cell cytotoxicity, (**d**) mRNA expression in NK cells, and (**e**) relative band intensity. Different letters indicate significant differences (*p* < 0.05) between MPF2 and its derivatives at each cytokine.

The effect of MPF2 and its derivatives (HS1, 2, 3 and OS1, 2, 3) on the proliferation of NK cells after 24 h of treatment is shown in Figure 7a. The level of NK cells proliferation was minimally influenced by the treatment of MPF2 and its derivatives, indicating that these samples were non-toxic to the NK cells at the tested concentration. The effect of MPF2 and its derivatives on NK cell cytotoxicity was tested against HepG2 cells at the effector with a target ratio of 30:1 (Figure 7b). As for the medium, NK cells showed 30.3% cytotoxicity against HepG2 cells, implying that NK cells themselves exhibited some direct cytotoxicity on the target cancer cells. When cells were treated with OS1, 2, and 3, the cytotoxicity of NK cells markedly increased to 50.8%, 54.7%, and 47.2%. This indicated that the OS1, 2, and 3 treatments effectively improved NK cell cytotoxicity. However, NK cell cytotoxicity observed by the HS1, 2, and 3 treatments were the same as MPF2. In contrast, cytotoxicity increased from 50.8% to 54.7% in OS1 and OS2 treatment. Although the level of NK cell cytotoxicity was less than 61.2% with OS2 treatment, the cytotoxicity was comparable to that exhibited by the positive control, 5-Fu (61.2%, 10 µg/mL). A similar level of NK cell cytotoxicity was reported in our previous study [50,61]. For OS1 and OS2 treatment in this study, there might be ranges of DS optimum on OS1 and OS2 for the RAW264.7 cell activation. As the percentage of improvement in NK cell activity reviews, the improvement in NK cell activity is on par with MPF2. The over-sulphation (OS1, 2, 3) showed better activity in the percentages of 10.7%, 19.3%, and 2.78%, but little was observed in HS1, 2, 3 (Figure 7c).

Similar findings were reported by Zhao et al. that sulphated polysaccharides extracted from red seaweed, *Polysiphonia senticulosa*, effectively enhanced immune function by activating the NK cells [62]. It was reported that grafted polysaccharides by over-sulphation to polysaccharides with a higher DS had better biological activity. Wang et al. [40] reported that the sulphated derivatives exhibited excellent antitumor activity when its DS was within the scope of 0.81–1.29. In addition, sulphated polysaccharides SALP-1 (DS, 0.48) and SALP-2 (DS, 0.73) extracted from *Acanthopanax leucorrhizu* significantly increased the DPPH and OH radical antioxidant activities compared to native polysaccharides (ALP) [63]. This result suggested that over-sulphation might be beneficial for the improvement of NK-92 cell activation with the optimum range of DS. However, the level of NK cell cytotoxicity observed with HS1, 2, and 3 treatments did not change. This implied that the low Mw of MPF2 did not play a critical role in the enhancement of NK-92 cell activation. 

The level of gene expressions such as IFN-γ, Granzyme-B and Perforin, NKp30, and FasL is shown in Figure 7d,e. The expression of IFN-γ mRNA was increased (up-regulated 2.0-fold compared to the medium) by the treatment of MPF2. Similarly, the mRNA levels of granzyme-B and perforin were also up-regulated by MPF2, showing a 2.5- and 1.3-fold enhancement. The mRNA expression of the surface-activating receptor, NKp30, exhibited significant up-regulation (3.0-fold) after the treatment of MPF2. In addition, the mRNA expression of apoptosis-inducing ligand FasL was notably up-regulated (1.7-fold) by the MPF2 treatment as well. Revealed by the level of NK cell cytotoxicity (Figure 7b), the gene expression was more significant from the OS2 treatment than MPF2, indicating that OS2 may possess a better capacity to activate NK cells. This compares to RAW264.7 cell activation, in which the Mw of the polysaccharides were important factors (Figure 4b). NK cell activation seemed tightly related to the sulphate content of the polysaccharides; therefore, when MPF2 possess sulphates, its NK cell signalling capacity is considerably increased. This could potentially be due to the increased interaction between OS2 and the NK cell surface receptors, which are different from those of RAW264.7 cells. Wang et al. [64] also reported the presence of negative charges of sulphated groups on the structure of polysaccharides, which can increase the binding capacity of immune cells through the electrostatic interaction and various chemical bonding resulting in improved bioactivity [39].

**Figure 8:**
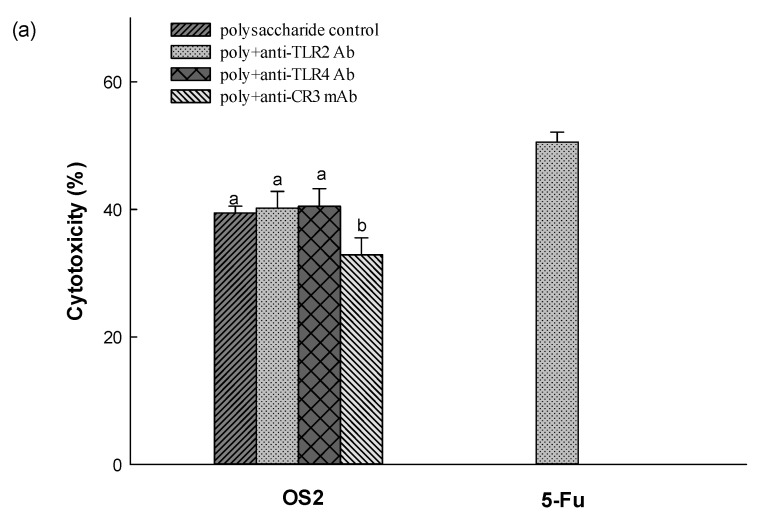

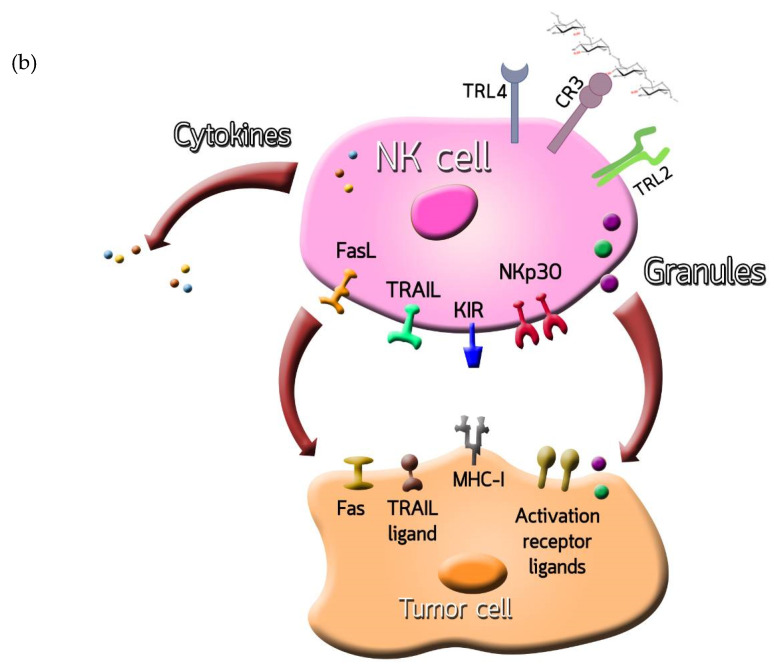
Effect of OS2 cytotoxicity against HepG2 cells in antibody neutralization: (**a**) cytotoxicity against HepG2 cells and, (**b**) a schematic illustration for the interaction of OS2 with CR3. Different letters indicate significant differences (*p* < 0.05) between polysaccharide control (OS2), and OS2 + anti-TLR2 Ab, OS2 + anti-TLR4 Ab, and OS2 + anti-CR3 Ab.

The possible interaction of NK-92 cell activation by MPF2 and its derivatives (HS3 and OS2) were examined by antibody neutralization. As shown in Figure 5c, the major surface receptors in RAW264.7 cells bound by polysaccharides are TLR4. However, among the various pattern recognition molecules (TLR2 and TLR4 and CR3) expressed on the NK cell surface, the anti-CR3 antibody treatment failed to enhance the cytotoxicity of MPF2 and its derivatives (Figure 8a,b), suggesting that MPF2 and its derivatives (OS2) may promote NK cell activity via a CR3-mediated signalling pathway. Huang et al., inferred that the sulphate content binding to polysaccharides can activate NK cells via the CR3 receptor [65]. Overall results suggest that the hydrolysed polysaccharides of MPF2 from *A. hemibapha* may have a better interaction with the surface receptors on RAW264.7 cells, but the interaction with those on NK cells could be promoted by the over-sulphation of MPF2. The enhanced interaction with the surface receptors on RAW264.7 cells and NK cells with polysaccharide derivatives was also observed, resulting in improved cell cytotoxicity.

## 4. Conclusions

In this study, a water-soluble polysaccharide was extracted from the fruiting bodies of *A. hemibapha* using the hot water extraction method. MPF2, an immunostimulant fraction, was sulphated by over-sulphation, and the Mw was lowered by the hydrolysis method. The effects of Mw and sulphate obtained from *A. hemibapha* subspecies *javanica* (Corner and Bas) on the immunomodulation were systematically investigated through the NO production in RAW264.7 cells and NK cell cytotoxicity against HepG2 cells. Lower Mw is the main factor for stimulating the RAW264.7 cells in order to produce pro-inflammatory mediators including NO, TNF-α, IL-1β, IL-6, and IL-10 through the nuclear factor kappa-B (NF-κB) and mitogen-activated protein kinases (MAPK) pathways. Lowering the Mw could be activated through the TLR4 signalling pathway in the macrophage cell receptors. The presence of sulphate groups in MPF2 was influential for NK-92 cell activation by inducing cytotoxicity in HepG2 cells through the expression of IFN-γ, Granzyme-B, NKp30, and FasL. The sulphated derivatives improved NK cell activation, therefore concluding that the chemical and structural modifications of the MPF2 from *A. hemibapha* subspecies *javanica* (Corner and Bas) could be used for functional food applications, nutraceuticals, and a source of development for new drugs.

## Figures and Tables

**Table 1:** The sequences of primers used for RT-PCR.

		Primer Sequences (5′→3′)
iNOS	Forward	CTGCAGCACTTGGATCAGGAACCTG
	Reverse	GGGAGTAGC CTGTGTGCACCTGGAA
IL-1β	Forward	ATGGCAACTATTCCAGAACTCAACT
	Reverse	CAGGACAGGTATAGATTCTTTCCTTT
TNF-α	Forward	AGGTTCTGTCCCTTTCACTCACTG
	Reverse	AGAAGACCTGGGAGTCAAGGTA
IL-6	Forward	TTCCTCTCTGCAAGAGACT
	Reverse	TGTATCTCTCTGAAGGACT
IL-10	Forward	TACCTGGTAGAAGTGATGCC
	Reverse	CATCATGTATGCTTCTATGC
β-actin	Forward	ATGTGCAAAAAGCTGGCTTTG
	Reverse	ATTTGTGGTGGATGATGGAGG
IFN-γ	Forward	GATGCTCTTCGACCTCGAAACAGCAT
	Reverse	ATGAAATATACAAGTTATAATCTTGGCTTT
Granzyme-B	Forward	AGATCGAAAGTGCGAATCTGA
	Reverse	TTCGTCCATAGGAGACAATGC
Perforin	Forward	AGTCCTCCACCTCGTTGTCCGTGA
	Reverse	AAAGTCAGCTCCACTGAAGCTGTG
NKp30	Forward	TCTATTACCAGGGCAAATGTGAAGT
	Reverse	GTCACTGGGGTCTAGAATCACTCAT
FasL	Forward	CCAGAGAGAGCTCAGATACGTTGAC
	Reverse	ATGTTTCAGCTCTTCCACCTACAGA
β-actin	Forward	CATCTCTTGCTCGAAGTCCA
	Reverse	ATCATGTTTGAGACCTTCAACA

**Table 2:** Preparation conditions for over-sulphation (OS1, OS2, and OS3) and sulphate content of MPF2 and its derivatives from *A. hemibapha*.

Sample	Temperature (°C)	Reaction Time (h)	DS	Sulphate Content (%)
MPF2	-	-	-	9.30 ± 0.20 ^d^
OS1	80	2	0.72	9.85 ± 0.05 ^c^
OS2	80	4	0.89	11.3 ± 0.21 ^b^
OS3	80	6	1.31	14.2 ± 0.21 ^a^

Different letters indicate significant differences (*p* < 0.05) among the MPF2 and its derivatives.

**Table 3:** Preparation conditions for acid hydrolysis (HS1, HS2, and HS3) and molecular weight of MPF2 and its derivatives from *A. hemibapha*.

Sample	Temperature (°C)	Reaction Time (min)	Yield (%)	Mw (×10^3^ g/mol)
MPF2	-	-	-	104.0 ± 13.1 ^a^
HS1	100	5	80	88.1 ± 3.52 ^b^
HS2	100	10	81	50.7 ± 5.20 ^c^
HS3	100	15	77	32.8 ± 3.87 ^d^

Different letters indicate significant differences (*p* < 0.05) among MPF2 and its derivatives.

## Data Availability

Any additional data can be made available upon request to the corresponding author.

## Supplementary Materials

The following are available online at www.mdpi.com/article/10.3390/jof7100847/s1, Table S1: Enhanced chemiluminescence (ECL) kit, and its instructions.
