# Supplementary material for: Sulphation and Hydrolysis Improvements of Bioactivities, and Immuno-Modulatory Properties of Edible Amanita hemibapha Subspecies javanica (Corner and Bas) Mucilage Polysaccharide as a Potential in Personalized Functional Foods
Source: J Fungi (Basel). 2021 Oct 10;7(10):847. doi: 10.3390/jof7100847 (PMC8540376; doi:10.3390/jof7100847)
Supplement: Supplementary file 1 [file jof-07-00847-s001.zip › jof-1402744-supplementary.pdf]

Table S1. Enhanced chemiluminescence (ECL) kit and its instructions.

**ECL kit instructions**
1. Prepare working solution of western blot detection by mix the solution A and B in the ratio of 40:1 (390 μl of Solution A + 10 μl of Solution B)
2. Incubate the membrane with detection buffer for 5 min
3. Drain excess reagent and cover the blot with clear film
4. Detect the signal by ChemiDocTM imaging system

- The signals were measured using Pierce®ECL Plus Western Blotting Substrate (Thermo Scientific, Waltham, MA, USA).
